# Supplementary figures and images for: Comprehensive Characterization of a Streptococcus agalactiae Phage Isolated from a Tilapia Farm in Selangor, Malaysia, and Its Potential for Phage Therapy
Source: Pharmaceuticals (Basel). 2023 May 5;16(5):698. doi: 10.3390/ph16050698 (PMC10221047; doi:10.3390/ph16050698)

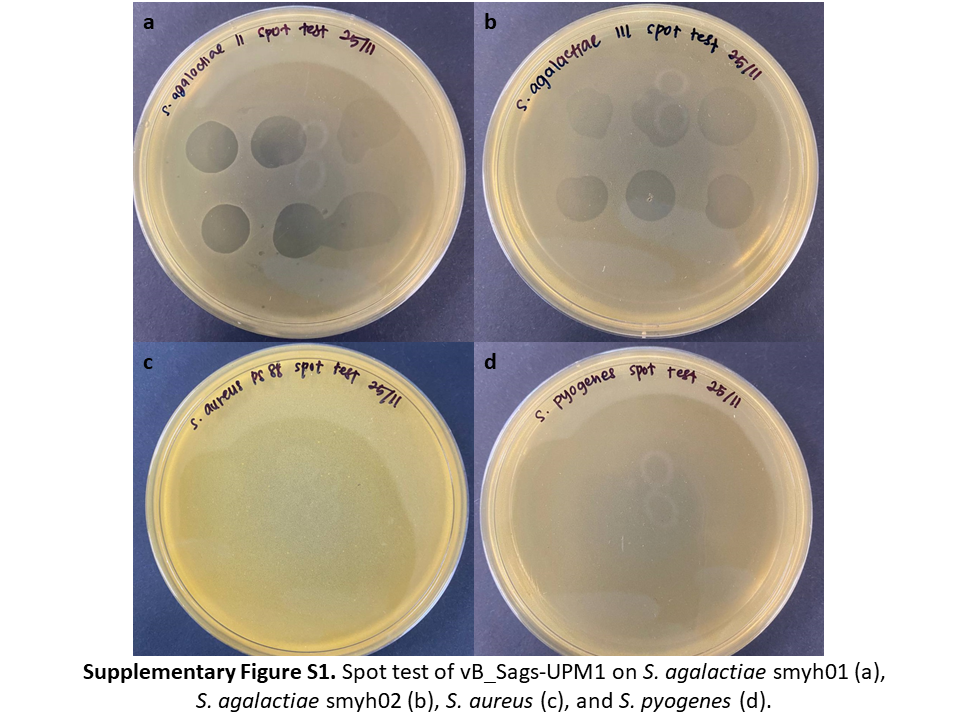

Supplement: Supplementary file 1 [file pharmaceuticals-16-00698-s001.zip › Supplementary Figure S1.tif]

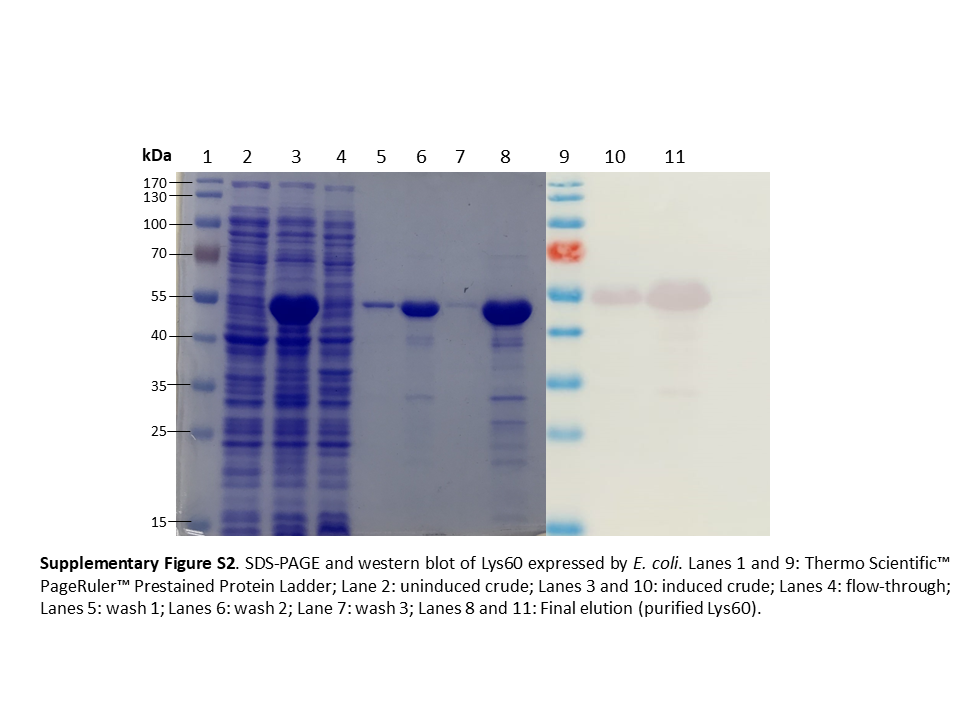

Supplement: Supplementary file 1 [file pharmaceuticals-16-00698-s001.zip › Supplementary Figure S2.tif]
